# Evaluation of Anterior Segment Parameters and Possible Influencing Factors in Normal Subjects Using a Dual Scheimpflug Analyzer

**DOI:** 10.1371/journal.pone.0097913

**Published:** 2014-05-16

**Authors:** Xiaogang Wang, Jing Dong, Qiang Wu

**Affiliations:** 1 Affiliated Sixth People's Hospital *Shanghai Jiao Tong University*, Shanghai, P.R. China; 2 Shanxi Eye Hospital, Taiyuan, Shanxi, P.R. China; 3 The First Hospital of *Shanxi Medical University*, Shanxi, P.R. China; Medical University Graz, Austria

## Abstract

**Purpose:**

To investigate normal anterior segment parameters and analyze the possible influencing factors using a dual Scheimpflug system.

**Setting:**

Department of Ophthalmology, Affiliated Sixth People's Hospital *Shanghai Jiao Tong University*, Shanghai, China.

**Design:**

A prospective observational case series.

**Methods:**

A total of 153 normal subjects (153 eyes) were studied. The anterior segment parameters, including the central corneal thickness (CCT), anterior chamber depth (ACD), pupil diameter (PD), keratoconus prediction index (KPI), simulated keratometry (SimK) values, anterior instantaneous curvature (AIC), posterior axial curvature (PAC), corneal eccentricity, total corneal power (TCP), axial curvature (AC), total corneal wavefront (TCW), high order aberration (HOA), and spherical aberration (SA), were determined using a dual Scheimpflug analyzer.

**Results:**

The CCT and ACD were both negatively correlated with age (r = −0.203, p = 0.012; r = −0.589, p<0.001). There was no significant difference in the refractive indices of AIC and SimK. Compared with the negative correlation of HOA and SA (r = −0.358, p<0.001), a positive correlation was found between TCW and HOA (r = 0.561, p<0.001). Unlike the decreased tendency of AC, the TCP increased gradually from the center to the periphery in the central 8 mm diameter. TCP showed a significant correlation with AC in the analyzed area.

**Conclusions:**

AIC and SimK provide different information in clinic, but the refractive indices of them showed no difference in this healthy study population, and age should be considered when using CCT and ACD values.

## Introduction

Corneal refractive characterizations such as central corneal thickness (CCT), anterior curvature, posterior curvature, total corneal power (TCP), and total corneal wavefront (TCW) are useful in all types of refractive surgery, particularly laser assisted in situ keratomileusis (LASIK). [1]–[5] Moreover, understanding how keratorefractive surgery affects the corneal shape and structure is becoming increasingly important. Anterior chamber depth (ACD), defined as the distance from the tear film or corneal endothelium to the anterior surface of the lens, is one of the most important factors in intraocular lens calculation. Detailed posterior corneal curvature information is of benefit for the detection of mild keratectasia.

Corneal topography is a valuable tool for evaluating diseases related to corneal shape changes, especially for the diagnosis of keratoconus. Precise measurement of anterior segment parameters in normal and keratoconic corneas is extremely important for diagnosing and monitoring corneal-related diseases as well as for surgical planning.

The Galilei Dual-Scheimpflug analyzer (GSA) is a non-contact and high-precision optical system used to investigate corneal topography using a rotating Scheimpflug camera and a placid disk. It can reconstruct a three-dimensional image of the anterior segment and provide detailed information of the anterior and posterior surface of the cornea, in addition to the ACD from the corneal endothelium to the crystalline lens, by internal software. Furthermore, the relationships between potential influencing factors, such as age, gender, refractive error, corneal curvature, and anterior segment parameters, are still uncertain. [6]–[10] Therefore, the purpose of this prospective study was to observe corneal refractive status in normal subjects and to investigate the possible influence of some factors, such as age, gender, refractive error, and corneal curvature, using the GSA system.

## Materials and Methods

The pupil diameter (PD) and other parameters within the region of interest (ROI) of approximately 1–4 mm diameter, such as the simulated keratometry (SimK), anterior instantaneous curvature (AIC), posterior axial curvature (PAC), total corneal power (TCP), axial curvature (AC) of the central 8 mm diameter, and total corneal wavefront (TCW) in the ROI of approximately 6 mm diameter were all determined by the Galilei Dual-Scheimpflug analyzer (Ziemer Group, Port, Switzerland) after 5 minutes dark adaption. The detailed description of each parameter follows.

1. The SimK values are calculated using the keratometric index (KI, n = 1.3375) from the Placido topographer. This index is different from the actual corneal refractive index (n = 1.376) because it does not take into account the posterior surface parameters.
2. The AIC, which uses the same KI as the SimK values, is calculated by the actual curvature radius in the intersection of the corneal surface point.
3. The PAC, which uses the real refractive index of the cornea (n = 1.376) and aqueous (n = 1.336), is calculated by the axial length that runs perpendicular from the intersection point to the reference axis.
4. The TCP, which uses the actual refractive index of air (n = 1.0), cornea and aqueous, is calculated by ray-tracing through the anterior and posterior surface using Snell's Law. The TCP in the central 8 mm, which was divided into central (0–4 mm diameter), paracentral (4–7 mm diameter) and peripheral (7–8 mm diameter) zones, was assessed.
5. The AC within the central 8 mm, which was divided into central (0–4 mm diameter), paracentral (4–7 mm diameter) and peripheral (7–8 mm diameter) zones, was assessed.
6. The root mean square (RMS) of the wavefront, which was centered on the pupil and recorded the approximate central 6 mm diameter, was calculated from the front and back surface. It is shown as the RMS in microns and consists of three parts: RMS in total, RMS of high order aberration (HOA) and RMS of spherical aberration.
7. The eccentricity (ε^2^) was calculated from the central 8 mm diameter of the cornea. It is based on the mathematical description of an ellipse and it defines the shape of the cornea.
8. The keratoconus prediction index (KPI), which is potentially useful for the early detection of keratoconus, was calculated following discriminant analysis of SimK1, SimK2, the differential sector index, and the opposite sector index. [11]

Previous studies showed high repeatability and reproducibility of GSA[12]–[14], so a measurement was performed once in each eye by the same operator. Based on the minimal image quality requirement for motion compensation (85%), Placido (85%), Scheimpflug (90%) and motion distance (70%), the image overall quality higher than 95% was chosen for further analysis. The subjects were instructed to blink completely just before the measurement was taken. GSA is a dual Scheimpflug system that measures the ocular anterior segment from the anterior corneal surface to the posterior lens surface; any images located behind the iris are blocked by the iris pigments. All of the indexes were calculated by the Galilei software (Version 5.2.1). The intraocular pressure (IOP) was measured by a non-contact air-puff tonometer (Topcon CT-80; Topcon Corp., Tokyo, Japan). The axial eye length was measured using an IOL Master 500 (Zeiss, Oberkochen, Germany), and the objective total refraction was determined using an auto refractometer KR-8800 (Topcon, Tokyo, Japan).

### Subjects

The study included 153 normal subjects (58 males, 95 females), and written informed consent was obtained from all participants. For the subjects, who are less than 18 years old, the written informed consent forms was obtained from the guardians on the behalf of the children participants involved in this study. One eye from each subject was randomly selected for analysis. The normal volunteers were chosen randomly (every third subject from the Physical Examination Center at the Shanghai Sixth People's Hospital) to decrease the selection bias. Han-people account for more than 90% of the Chinese population, so the Han-Chinese participants were selected using the unique ethnicity information on the volunteers' identity cards. This helped eliminate any possible influence from different ethnic groups. Ethics committee approval was obtained from the Shanghai Clinical Research Center. The subjects' ages ranged from 12 to 85 years (mean 34±17 years). Eligible subjects had a normal ophthalmic examination that included the following: a best-corrected visual acuity of ≥20/40, a refractive error <5 diopter (D) spherical and <3 D cylinder, normal slit-lamp and fundoscopy examinations, an axial length <24.5 mm and an IOP <22 mmHg. [15] The exclusion criteria included all detectable ocular diseases, recent ocular surgery, wearing contact lenses, and the use of eye drops. To avoid any fluctuations in corneal thickness because of the time of day, the measurements were all made at noon.

### Statistics

Statistical analyses were performed using commercial software (SPSS ver. 13.0; SPSS Inc., Chicago, IL, USA). To compare the difference between AIC and SimK, an independent sample t-test was performed. Linear regression analysis was employed to investigate whether the measurements of CCT and ACD were affected by age and also to demonstrate the relationship of HOA versus TCW and SA versus HOA. Pearson correlation analysis was used to evaluate correlations between CCT and age, gender, mean TCP, ACD, mean refractive error and mean cylindrical refractive error, and also to evaluate correlations between TCP and AC in the central 8 mm diameters. A multivariate regression model was used to analyze the effect of age on ACD while controlling for several other independent variables, including gender and pupil diameter. All of the tests had a significance level of 0.05.

## Results

**Figure 1:**
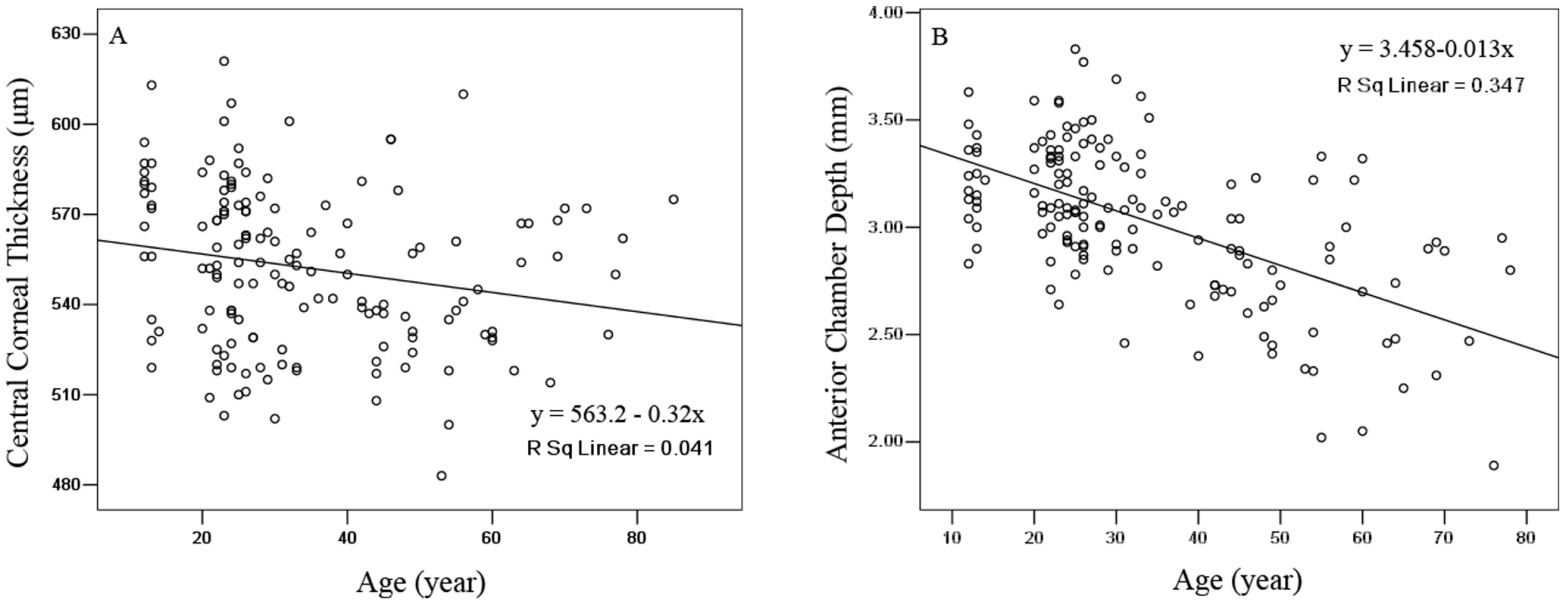
Scatter plot of (A) age against central corneal thickness, and (B) age against anterior chamber depth as measured by the Galilei Scheimpflug system. Line: univariate regression summarizing the relationship between the two variables.

A total of 153 subjects (153 eyes) were evaluated using GSA. The values for the anterior segment parameters are shown in Table 1. Corresponding to the values in Table 1, the SimK values in units of mm were 7.77±0.23 for average, 7.86±0.24 for flat, and 7.68±0.24 for steep; the AIC values were 7.81±0.24, 7.90±0.24, and 7.73±0.25; and the PAC values were 6.39±0.23, 6.56±0.24, and 6.22±0.25, respectively. There was no significant difference between AIC and SimK in each corresponding index (Table 2). Figure 1A shows the regression of CCT on age. A linear model best demonstrated this relationship: CCT (µm)  = 563.2–0.32*age (r = −0.203, p = 0.012). According to this model, a 10-year increase in age results in an approximate 3.2 µm decrease in CCT. CCT was also negatively correlated with mean TCP (r = −0.172, p = 0.033) but not correlated with other indices such as gender, ACD, mean refractive error and mean cylindrical refractive error. ACD was positively correlated with gender (r = 0.254, p = 0.002) and PD (r = 0.409, p<0.001). Figure 1B shows the regression of ACD with age. A linear model best demonstrated this relationship: ACD (mm)  = 3.458–0.013*age (r = −0.589, p<0.001). According to this model, a 10-year increase in age results in an approximate 0.13 mm decrease in ACD.

**Table 1:** Normal values of anterior ocular parameters.

	M±SD	Minimum	Maximum
CCT (µm)	552±26	483	621
ACD (mm)	3.03±0.35	1.89	3.83
KPI (%)	2.8±4.5	0	21.9
PD (mm)	3.04±0.56	1.24	4.87
**SimK Values (D)**			
SimK average	43.47±1.29	39.43	47.17
SimK f	42.96±1.28	39.20	46.54
SimK s	43.99±1.38	39.66	47.81
Astigmatism	1.03±0.61	0.07	3.16
**Anterior Instantaneous Curvature (D)**			
K average	43.23±1.29	39.23	46.63
K f	42.74±1.28	39.09	46.00
Ks	43.72±1.37	39.36	47.26
Astigmatism	0.98±0.55	0.1	2.94
ε^2^	0.25±0.17	−1.06	0.61
**Posterior Axial Curvature (D)**			
K average	−6.27±0.23	−6.84	−5.67
K f	−6.10±0.22	−6.66	−5.50
Ks	−6.44±0.26	−7.02	−5.73
astigmatism	−0.34±0.14	−0.71	−0.02
ε^2^	0.31±0.38	−0.24	3.35
**Total Corneal Power (D)**			
Mean	41.77±1.27	37.81	45.44
Flat	41.31±1.26	37.51	44.90
Steep	42.24±1.34	38.10	45.98
Astigmatism	0.93±0.58	0.06	3.10
Central Avg. (0–4 mm diameter)	41.73±1.27	37.78	45.45
Paracentral Avg. (4–7 mm diameter)	42.25±1.34	38.33	45.85
Peripheral Avg. (7–8 mm diameter)	42.63±1.46	38.85	46.52
**Axial Curvature (D)**			
Central Avg. (0–4 mm diameter)	43.50±1.30	39.48	47.25
Paracentral Avg. (4–7 mm diameter)	42.98±1.28	39.05	46.28
Peripheral Avg. (7–8 mm diameter)	42.33±1.27	38.81	45.38
**RMS Wavefront (µm)**			
Total	1.33±0.87	0.37	7.95
HOA	0.61±0.33	0.18	3.17
Spherical Aberration	0.19±0.13	−0.45	0.80

Note: anterior chamber depth (ACD), average (Avg.), central corneal thickness (CCT), diopter (D), flat (f), high-order aberration (HOA), keratoconus prediction index (KPI), mean (M), pupil diameter (PD), root mean square (RMS), simulated keratometry (SimK), steep (s), standard deviation (SD),

**Table 2:** Difference between anterior instantaneous curvature (AIC) and simulated keratometry (SimK) values.

	AIC (n = 153; D)	SimK (n = 153; D)	*P* ^†^
Average	43.23±1.29	43.47±1.29	0.100
Flat	42.74±1.28	42.96±1.28	0.144
Steep	43.72±1.37	43.99±1.38	0.085
Astigmatism	0.98±0.55	1.03±0.61	0.399

Note: diopter (D); † Two-tailed independent sample t-test.

**Figure 2:**
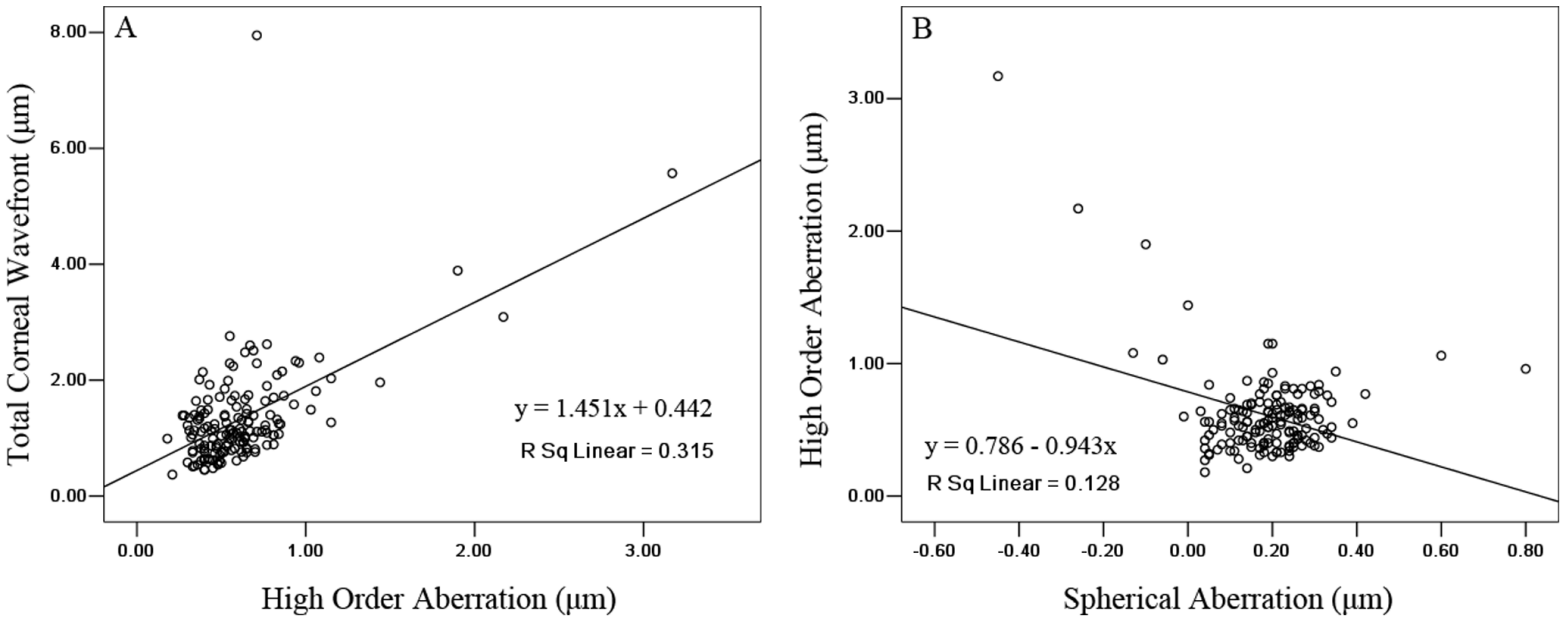
Scatter plots of (A) high order aberration against total corneal wavefront, and (B) spherical aberration against high order aberration as measured by the Galilei Scheimpflug system. Lines: univariate regression summarizing the relationship between the two variables.

HOA was positively correlated with TCW (r = 0.561, p<0.001) and Figure 2A shows the regression of TCW with HOA. Conversely, corneal SA, as an important part of HOA, was negatively correlated with HOA (r = −0.358, p<0.001) and Figure 2B shows the regression of HOA with SA.

Compared with the decreased tendency of AC, the TCP increased gradually from the center to the periphery in the central 8 mm diameter (Table 1). Moreover, TCP was positively correlated with AC in the corresponding area (Table 3).

**Table 3:** Correlations between total corneal power (TCP) and axial curvature (AC) of the central cornea of 8 mm diameter.

	TCP & AC		
	0–4 mm diameter	4–7 mm diameter	7–8 mm diameter
r	0.994	0.991	0.982
p	.000	.000	.000

In the multivariate analysis where the dependent variable was ACD (Table 4), age was the dominant explanatory variable, accounting for approximately 35% of the variance (R2). Gender and PD did not account as much as age when grouped with age in the multivariate models. This suggests that age is a relatively strong indicator of changes in ACD.

**Table 4:** Multivariate regression model to predict anterior chamber depth associated with age and gender in normal subjects.

Model	Variable 1			Variable 2			Total R2
	Parameter	Slope (P-value)	R2	Parameter	Slope (P-value)	R2	
1	Age	−0.013 (.000)	0.347				
2	Age	−0.013 (.000)	0.347	Gender	0.201 (.000)	0.077	0.424
3	Age	−0.011 (.000)	0.347	Pupil diameter	0.112 (0.016)	0.025	0.372

## Discussion

Corneal ectasia is a well-recognized complication of refractive surgery, and posterior corneal elevation is an early presenting sign for keratoconus; therefore, it is essential to evaluate posterior corneal curvature in every refractive surgery candidate. With the help of non-invasive imaging techniques, the ocular structure can be reconstructed and observed clearly in higher resolution *in vivo*. Compared with ultrasound, Orbscan and Pentcam, GSA can produce a three-dimensional image of the anterior segment using the double Scheimpflug-Placido imaging technique.

There are a number of reports or evaluations of CCT values using different tools, such as slit-lamp-based, specular microscope-based, other optical/laser-based, or ultrasound-based measurements. According to a study by Mishima et al, the normal range of human CCT values should be between 500 and 570 µm, which is obviously different from the range of 700 to 1000 µm used pre-1950s. [16], [17] This may be largely attributed to improvements in the accuracy and precision of the measurement tools. Compared to recent GSA studies in normal subjects, CCT values in this study were similar. A mean value of 549.2±30.5 µm for 77 normal eyes was reported by Zaina, which is thicker than the 541.27±30.07 µm for 92 eyes in a study by Ladi et al, but thinner than the 560.57±29.10 µm reported for 47 eyes by Hosseini et al. [18]–[20] A number of factors may affect CCT, such as age, race, gender, refractive error, and corneal curvature. [8]–[10] In our study, we found that CCT decreased approximately 3.2 µm for each decade of increase in age. Similarly, several investigators reported a significant rate of decrease in CCT with age, but the decreased rate showed little difference. [21], [22] However, some studies have found that age does not affect CCT. [8], [23] Gender, ACD, mean refractive error and mean cylindrical refractive error had no significant effect on CCT, consistent with some previous reports. [8]–[10], [23]

Precise evaluation of ACD is important for surgical planning and follow-up in glaucoma and intraocular lens implantation. The ACD value of normal subjects in this study was a slightly deeper than the ∼2.8 mm measured with the Orbscan system and a slightly shallower than the ∼3.3 mm measured by the Pentacam system and anterior segment optical coherence tomography. [24], [25] This may be due to different races and accommodation status in the studies. ACD was correlated with age, gender and PD, in accordance with some previous studies. [6], [7], [24] A multiple regression model (Table 4) suggests that age is the main factor affecting ACD compared to gender and PD.

We obtained mean anterior and posterior corneal curvature values similar to a previous study that used Pentacam; in that study, values of 7.81±0.28 and 6.40±0.24 mm for the mean anterior and posterior corneal curvature, respectively, were obtained for the control group. [26] Moreover, our SimK values were similar to those of the study designed by Savini et al using Pentacam and videokeratography. [27] Because the same simulated KI (n = 1.3375) was used for the AIC and SimK calculation, it is easy to understand the consistency between AIC and SimK values. However, we should notice the difference between instantaneous curvature and the AC. Compared to a different calculation method for instantaneous curvature, AC is very dependent on the position of the reference axis. Instantaneous curvature can give a more detailed description of local curvature, but it may be very noisy. Although AC can give a more global description of shape, it will underestimate areas of relative higher curvature and overestimate areas of relative lower curvature. [28], [29]

The TCP values of the central 8 mm diameter were lower than in the Wang et al study, who reported values of 43.36±1.37, 44.05±1.51, and 44.30±1.67 D for the central, paracentral and peripheral zones in 20 normal eyes. [14] However, the two studies showed a similar increasing tendency for the TCP values of the central area. The K average and astigmatism values of PAC were similar with the Wang et al study, but lower than their results. [14] The SA and HOA all showed different values, although we used the same equipment for analysis. The different values may be due to different sample sizes and human races.

The KPI values in this study were mostly clustered around 0% and the mean value was less than 3%. A KPI with a range from 0–100% can indicate moderate and severe keratoconus, but it cannot distinguish them clearly, so its role in showing the degree of corneal asymmetry may be limited. [11], [30] Corneal eccentricity is an indicator of corneal asphericity. In this study, the anterior and posterior mean eccentricity values were similar (0.27±0.63) as measured by the Pentacam. [31] Compared to Orbscan, the GSA system can simultaneously provide an accurate analysis of the anterior and posterior corneal surfaces with the help of the Scheimpflug technique. The mean anterior and posterior corneal radii were much closer to the values (7.81±0.28 and 6.40±0.24 mm, respectively) in the Camellin et al study, that were measured using Pentacam in 71 eyes that had not been operated on. [26]

Wavefront technology may be a useful adjunct to topography for keratoconus diagnosis. [32]–[34] The normal eyes in our study showed that the TCW was positively correlated with corneal HOA and that HOA as negatively correlated with corneal SA. This may be helpful for whole ocular wavefront analysis in the future. However, various factors may add noise to the corneal wavefront measurement, such as variation in the location of the pupil and fluctuations in the tear film measurement. Several studies have shown that unstable tear films will increase irregular astigmatism and bad optical quality. [35]–[38] Moreover, HOA changed dynamically with blinking, even in normal subjects. [39]

The limitations of this study are that the refractive values and correlations were evaluated only in normal Chinese corneas and all of the measured values were not compared with those of other topographers. Therefore, further studies with multiple races, disease status, and other topographers are needed.

In conclusion, AIC and SimK provide different information in clinic, but the refractive indices of them showed no difference in this healthy study population, and age should be considered when using CCT and ACD values.
